# Scalp psoriasis: a rare clinical image

**DOI:** 10.11604/pamj.2022.42.265.36337

**Published:** 2022-08-10

**Authors:** Praveen Nandal, Sadhana Misar Wajpeyi

**Affiliations:** 1 Department of Kayachikitsa, Mahatma Gandhi Ayurveda College, Hospital and Research Centre, Salod (Hirapur), Datta Meghe Institute of Medical Sciences (DU), Sawangi, Wardha, India

**Keywords:** Scalp psoriasis, auto-immune disease, therapeutic purification, Ekkushtha

## Image in medicine

Psoriasis is a skin condition that affects about 2-3 percent of people worldwide, according to the National Psoriasis Foundation. But scalp psoriasis occurs in rare conditions. Scalp psoriasis is a long-lasting autoimmune disease having thick scales on clearly-defined, erythematous skin. Frequently, the scales are silvery white. The hairline may be partially covered by psoriasis. Even though the hair frequently serves as an effective covering for scalp psoriasis, the flaking of the scales and excessive 'dandruff' make it a common cause of social disgrace. Scalp psoriasis might be asymptomatic or can cause severe itching. Even though its severity frequently changes over the course of several years, it typically persists. The line of treatment for scalp soriasis includes topical therapy, intralesional therapy, systemic therapy, and photochemotherapy. According to ayurved, all skin disorders are included under kustha disease. Scalp psoriasis can be considered Ekkustha which is one of the types of kustha disease. A 26-year-old male student came with complaints of a red, scaly patch with itching on the lower occipital and neck region for 4-5 months. On clinical examination candle, grease sign, and auspitz sign were positive with normal blood count and blood glucose level. From this, it was diagnosed as scalp psoriasis as shown in the image. Therapeutic purification therapy and the palliative treatment mentioned in Ayurveda were given for 2 months. After 2 months of follow-up, complete remission was observed. This image is useful for differential diagnosis between seborrheic dermatitis, lichen planopilaris, and scalp psoriasis.

**Figure 1:**
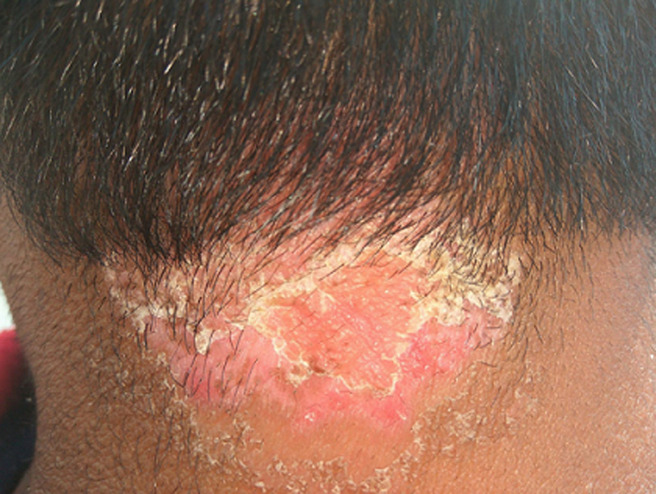
scalp psoriasis

